# Supplementary material for: A fundamental viewpoint on the hydrogen spillover phenomenon of electrocatalytic hydrogen evolution
Source: Nat Commun. 2021 Jun 9;12:3502. doi: 10.1038/s41467-021-23750-4 (PMC8190308; doi:10.1038/s41467-021-23750-4)
Supplement: Supplementary file 1 — Supplementary Information [file 41467_2021_23750_MOESM1_ESM.pdf]

Supplementary Information for

A fundamental viewpoint on the hydrogen spillover phenomenon of electrocatalytic hydrogen evolution

Jiayuan Li[1], Jun Hu[2,*], Mingkai Zhang[3], Wangyan Gou[1], Sai Zhang[1], Zhong Chen[4], Yongquan Qu[1,3,*] and Yuanyuan Ma[1,*]

[1] Key Laboratory of Special Functional and Smart Polymer Materials of Ministry of Industry and Information Technology, School of Chemistry and Chemical Engineering, Northwestern Polytechnical University, Xi'an 710072, China
[2] School of Chemical Engineering, Northwest University, Xi'an, P. R. China 710069
[3] Frontier Institute of Science and Technology, Xi'an Jiaotong University, Xi'an, 710049, China.
[4] School of Materials Science and Engineering, Nanyang Technological University, 50 Nanyang Avenue, Singapore 639798

Table of content:

Supplementary Methods: Page 1-2
Supplementary Figures 1-26 Page 3-17
Supplementary Tables 1-7 Page 18-25
Supplementary References 1-47 Page 26-29

Supplementary Methods:

Synthesis of Pt/rGO and Pt_2Ir_1/rGO benchmark catalysts: Such catalysts here were synthesized via the similar strategy mentioned above when replacing the CoP support by the commercial reduced graphene oxide (rGO, Sigma-Aldrich).

Theoretical derivation of the Tafel slope for the Pt_2Ir_1/CoP catalysts: Referring to the previous reports,[1,2] the HER pathway of Pt_2Ir_1/CoP is described by the following equations:

$$Pt_2Ir_1/CoP + H^+ + e^- \leftrightarrow H^*\text{–}Pt_2Ir_1/CoP \quad (1)$$

$$H^*\text{–}Pt_2Ir_1/CoP \leftrightarrow Pt_2Ir_1/CoP\text{–}H^* \quad (2)$$

$$Pt_2Ir_1/CoP\text{–}H^* + H^+ + e^- \leftrightarrow H_2 + Pt_2Ir_1/CoP \quad (3)$$

The reaction velocity of hydrogen evolution could be written as $r = k_3\theta_{CoP\text{–}H^*}C_H^{+}$, where r is the reaction rate; k is the rate constant; θ is the hydrogen coverage of on active sites; and C_H^{+} is the concentration of hydrogen ion.

In the steady state,

$$\frac{d\theta_{CoP-H^*}}{dt} = k_2\theta_{Pt_2Ir_1-H^*}(1-\theta_{CoP-H^*}) - k_{-2}\theta_{CoP-H^*}(1-\theta_{Pt_2Ir_1-H^*}) - k_3\theta_{CoP-H^*}C_{H^+}$$

$$\frac{d\theta_{Pt_2Ir_1-H^*}}{dt} = k_1(1-\theta_{Pt_2Ir_1-H^*})C_{H^+} - k_{-1}\theta_{Pt_2Ir_1-H^*} - k_{-2}\theta_{Pt_2Ir_1-H^*}(1-\theta_{CoP-H^*}) + k_{-2}\theta_{CoP-H^*}(1-\theta_{Pt_2Ir_1-H^*})$$

At the low overpotental,

$$\theta_{CoP-H^*} \approx \frac{k_2\theta_{Pt_2Ir_1-H^*}}{k_2\theta_{Pt_2Ir_1-H^*} + k_{-2} - k_{-2}\theta_{Pt_2Ir_1-H^*} + k_3C_{H^+}} \approx \frac{k_2}{k_{-2}}\theta_{Pt_2Ir_1-H^*}e^{-\frac{F\Delta\varphi}{RT}}$$

$$\theta_{Pt_2Ir_1-H^*} \approx \frac{k_1C_{H^+} + k_{-2}\theta_{CoP-H^*}}{k_1C_{H^+} + k_{-1} + k_2 + k_{-2}\theta_{CoP-H^*} - k_2\theta_{CoP-H^*}} \approx \frac{k_1}{k_{-1}}C_{H^+}e^{-\frac{F\Delta\varphi}{RT}}$$

Thus,

$$r \approx k_3\theta_{CoP-H^*}C_{H^+} = \frac{k_1k_2k_3}{k_{-1}k_{-2}}C_{H^+}^2e^{-\frac{(2+\alpha)F\Delta\varphi}{RT}} \quad (4)$$

And,

$$-j = Fr = \frac{k_1k_2k_3}{k_{-1}k_{-2}}FC_{H^+}^2e^{-\frac{(2+\alpha)F\Delta\varphi}{RT}}$$

$$lg(-j) = Constant + 2lgC_{H^+} - \frac{(2+\alpha)F}{2.303RT}\Delta\varphi \quad (5)$$

Therefore, the derived Tafel slope for Pt_2Ir_1/CoP catalysts is: $\frac{(2+\alpha)F}{2.303RT}$ = 0.023 V/dec (assuming α = 0.5, F is the Faraday constant, R is the Rydberg gas constant and T is the absolute temperature).

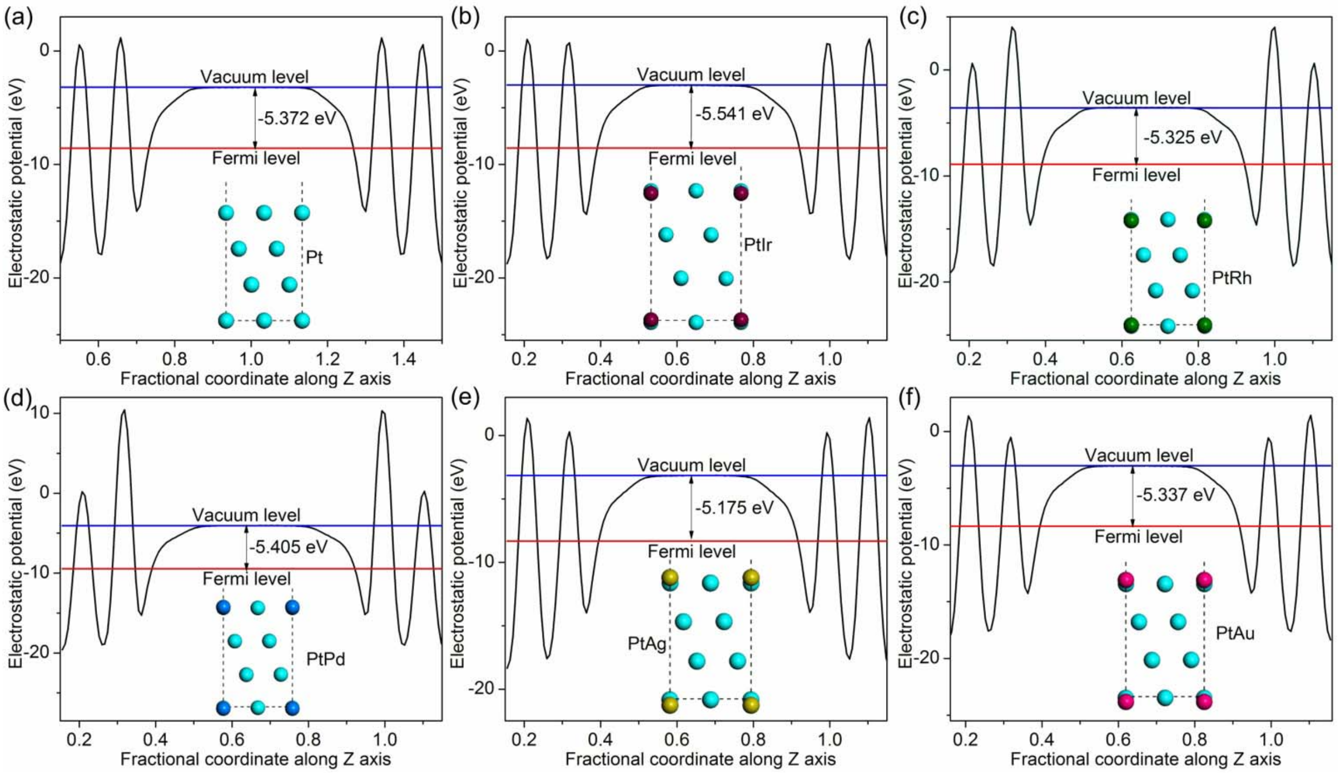

Supplementary Figure 1. Work function estimation for various PtM alloys by DFT calculations, where M = Pt **(a)**, Ir **(b)**, Rh **(c)**, Pd **(d)**, Ag **(e)** and Au **(f)**.

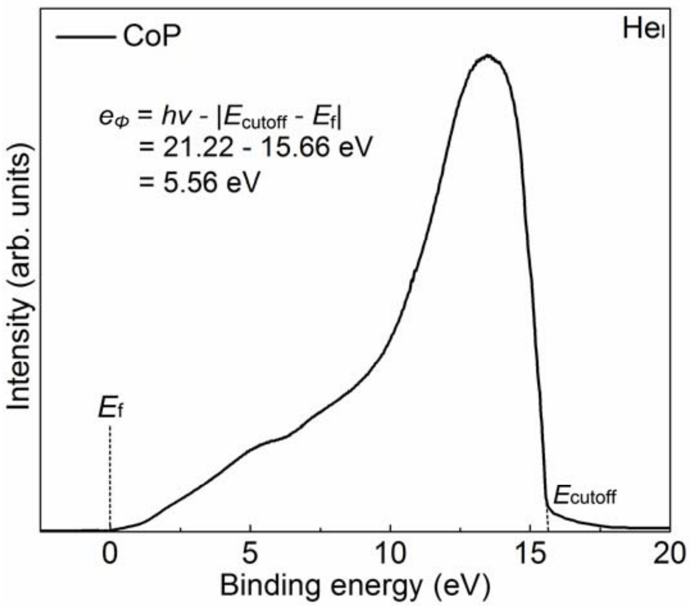

Supplementary Figure 2. Work function estimation for CoP by UPS analysis.

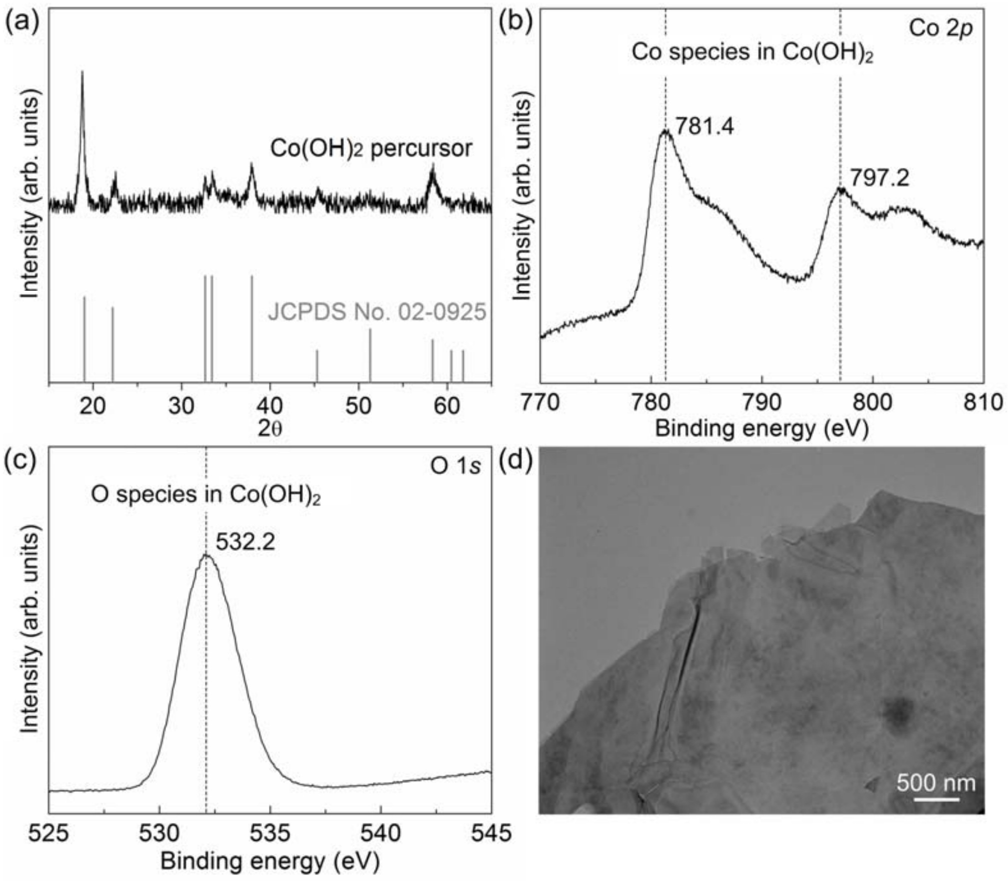

Supplementary Figure 3. Characterizations of $Co(OH)_2$ precursors. (a) XRD pattern. (b) High-resolution XPS spectra in Co 2*p* (b) and O 1*s* (c) region. (d) TEM image. XRD pattern of the product is consistent with that of $Co(OH)_2$ standard (JCPDS #02-0925), suggesting the successful synthesis of $Co(OH)_2$ precursors. In the high-resolution XPS of the product, the typical signals of Co and O species in $Co(OH)_2$ were identified, further confirming the formation of $Co(OH)_2$ precursors.[3] TEM image clearly demonstrates the morphology of nanosheet for the $Co(OH)_2$ precursors.

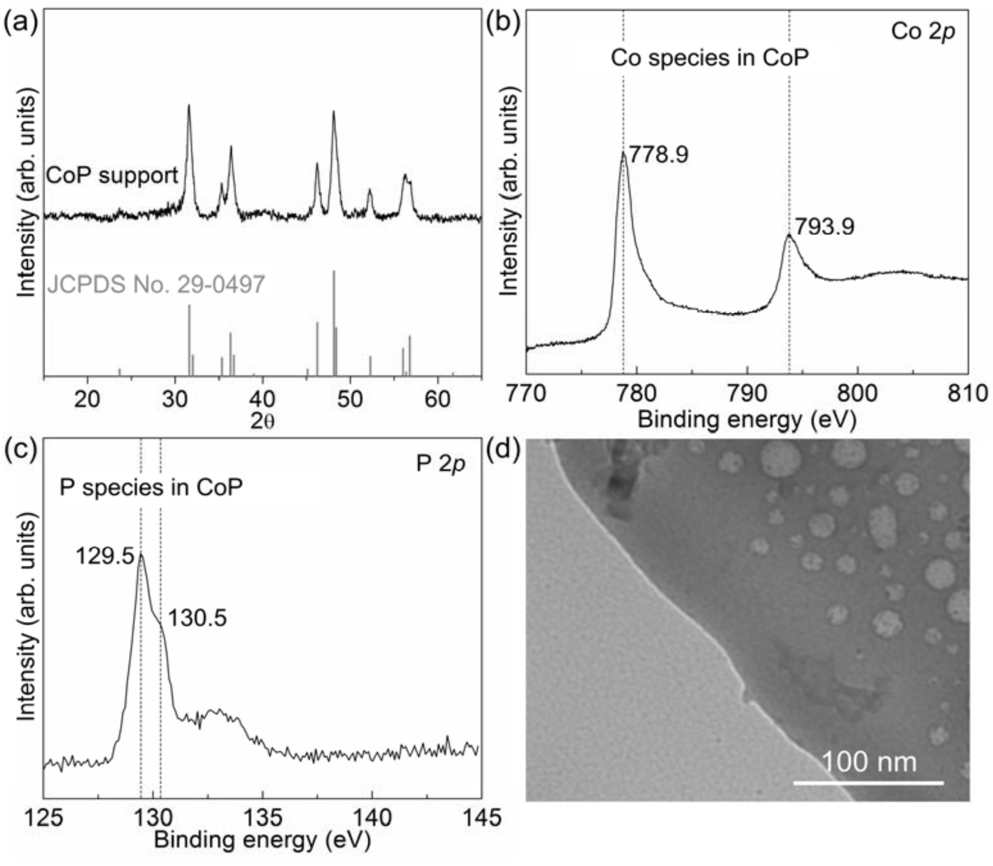

Supplementary Figure 4. Characterizations for CoP. (a) XRD pattern. (b) High-resolution XPS spectra in Co 2*p* (b) and P 2*p* (c) region. (d) TEM image. XRD pattern of the product is consistent with that of CoP standard (JCPDS # 29-0497), suggesting the successful synthesis of CoP. In the high-resolution XPS of the product, the typical signals of Co and P species in CoP were identified, further confirming the formation of CoP.[4] TEM image demonstrates the morphology of nanosheet for the CoP support.

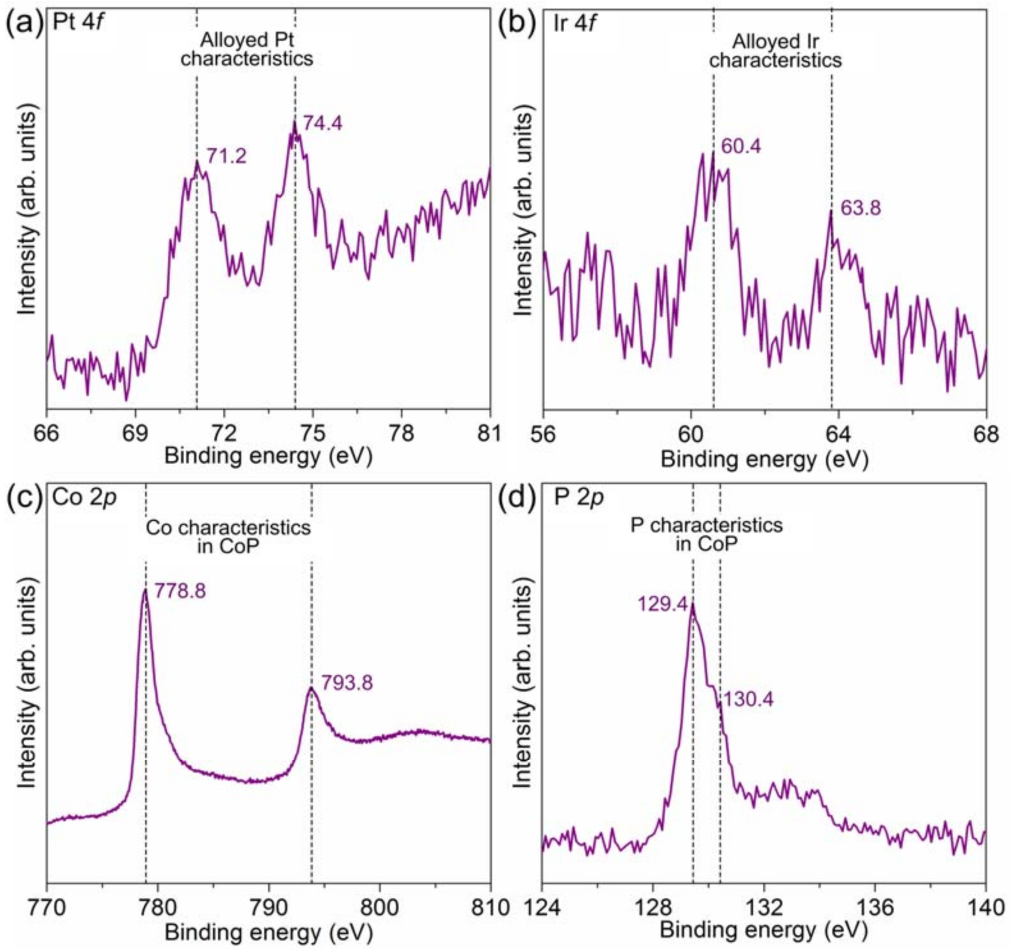

Supplementary Figure 5. High-resolution XPS spectra in **(a)** Pt 4*f*, **(b)** Ir 4*f*, **(c)** Co 2*p* and **(d)** P 2*p* region for the Pt_2Ir_1/CoP catalysts.

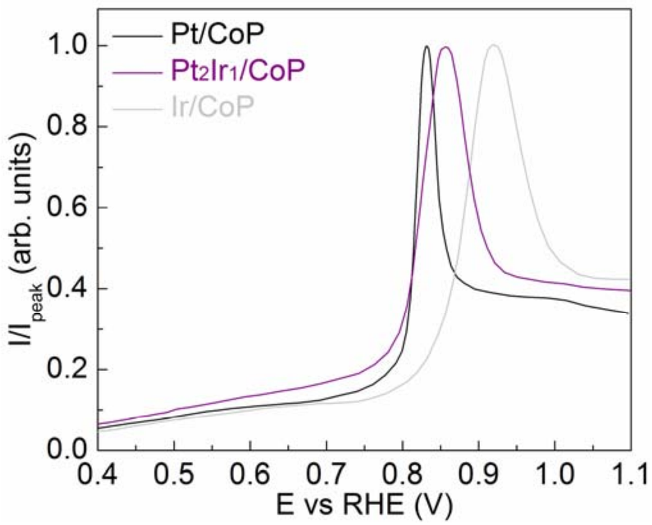

Supplementary Figure 6. CO stripping voltammetry of the Pt/CoP, Pt_2Ir_1/CoP and Ir/CoP catalysts in 0.5 M H_2SO_4 at scan rate of 20 mV/s. The CO was pre-adsorbed at 0.05 V vs. RHE for 15 min in each experiment. The CO stripping currents were normalized to each other. Thus, all of the peaks have the same magnitude to compare the CO stripping peak characteristics. For Pt/CoP and Ir/CoP, the CO stripping peaks were centered at 0.83 and 0.93 V *vs.* RHE, respectively, consistent with the previous report.[5] The much more positive CO stripping peak and much larger full width at half maximum (FWHM) for Ir/CoP over those for Pt/CoP could be explained as arising from the higher desorption activation energy of CO from Ir (22 kcal/mol) compared to that from Pt (13 kcal/mol).[6] If no Pt-Ir alloy is formed in the Pt_2Ir_1/CoP catalysts, the CO stripping voltammograms should show two clear peaks, centered at the potentials seen for the Pt/CoP and Ir/CoP catalysts alone.[5] Herein, the single CO stripping peak at 0.88 V vs RHE with moderate FWHM for the Pt_2Ir_1/CoP clearly demonstrated the formation of alloyed Pt_2Ir_1 with the regulated overall CO adsorption strength rather than the formation of only individual Pt and Ir.[5]

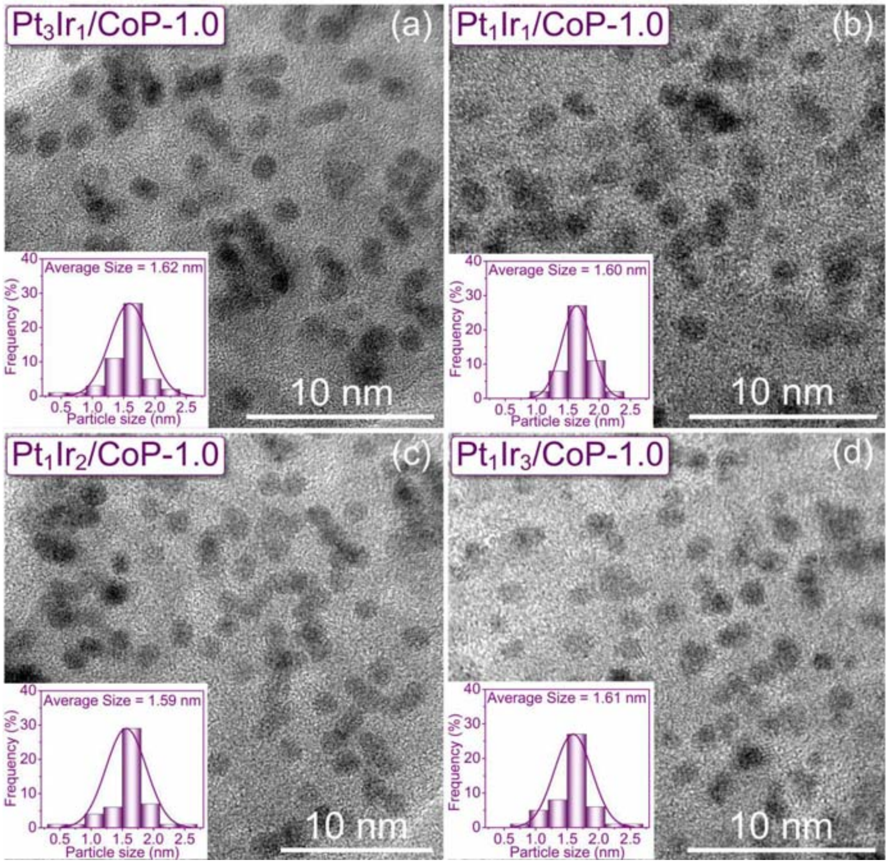

Supplementary Figure 7. TEM images for **(a)** Pt_3Ir_1/CoP, **(b)** Pt_1Ir_1/CoP, **(c)** Pt_1Ir_2/CoP and **(d)** Pt_1Ir_3/CoP at metal loading of 1.0 *wt*%. The insets show the size distributions of the loaded metal nano-particles.

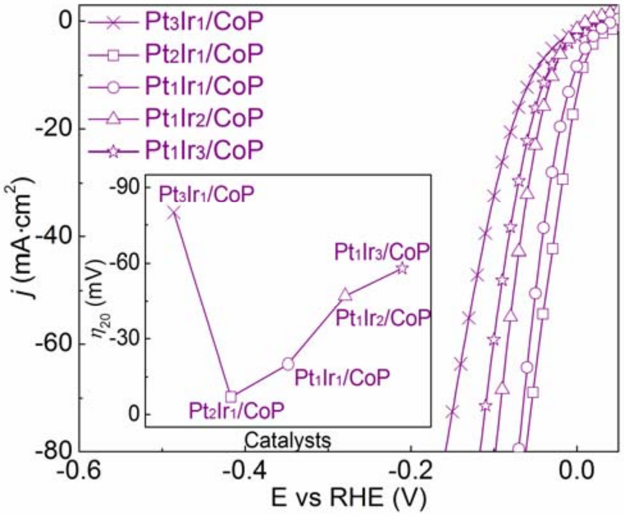

Supplementary Figure 8. HER performance for the PtIr/CoP catalysts with different molar ratio of Pt and Ir.

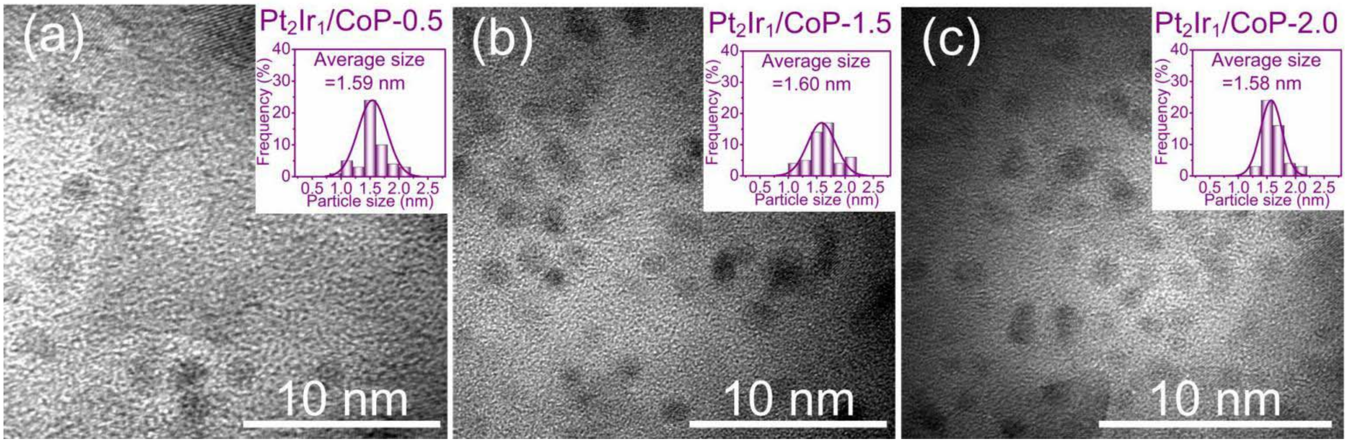

Supplementary Figure 9. TEM images for the Pt_2Ir_1/CoP catalysts at various metal loadings of **(a)** ~ 0.5 *wt*%, **(b)** ~ 1.5 *wt*% and **(c)** ~ 2.0 *wt*%. The insets show the size distributions of the loaded metal nanoparticles.

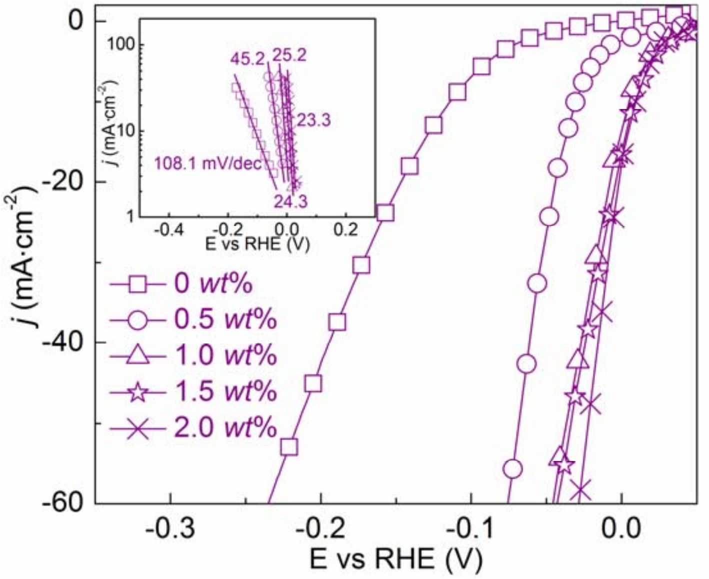

Supplementary Figure 10. HER performance for the Pt_2Ir_1/CoP catalysts at different metal loading.

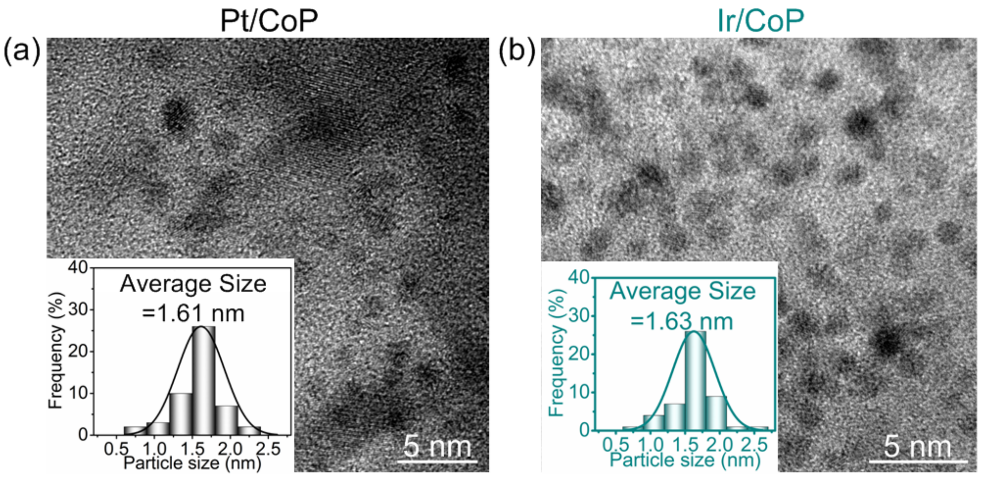

(c)

Catalyst	Catalyst mass (mg)	Constant volume (mL)	Measured metal content (ppm)	Metal loading (*wt. %*)
Pt/CoP	50	100	5.4 (Pt)	1.08 (Pt)
Ir/CoP	50	100	5.2 (Ir)	1.04 (Ir)

Supplementary Figure 11. TEM images for the **(a)** Pt/CoP and **(b)** Ir/CoP at metal loading of ~ 1.0 *wt*%. **(c)** ICP analysis for the Pt/CoP and Ir/CoP catalysts.

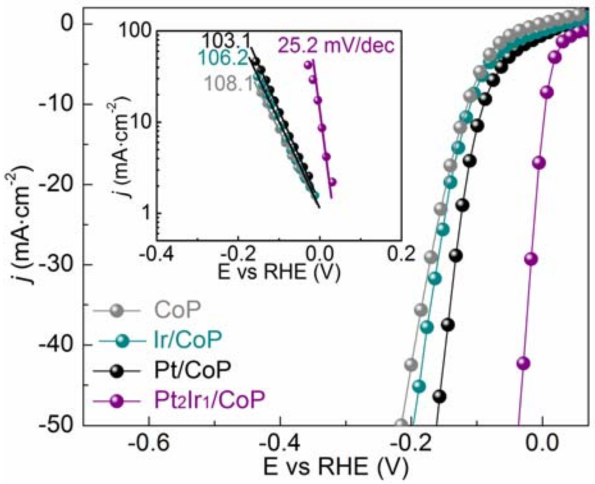

Supplementary Figure 12. HER performance for the Pt/CoP, Ir/CoP and Pt_2Ir_1/CoP catalysts.

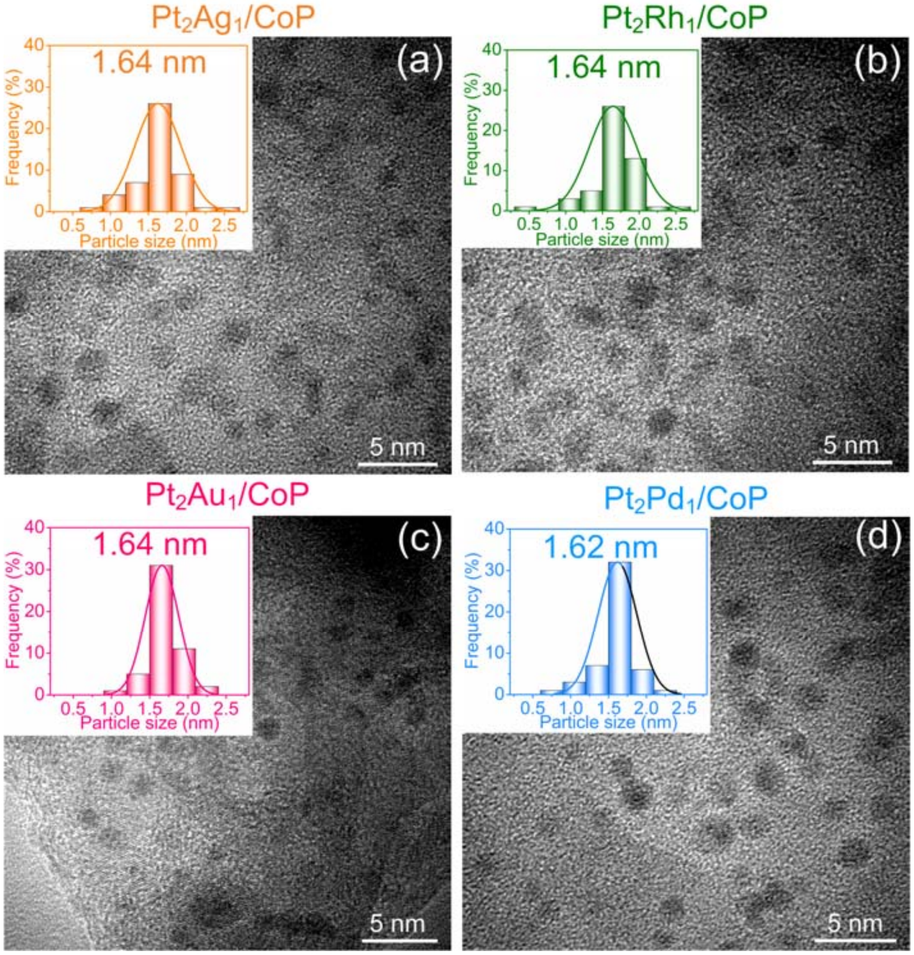

Supplementary Figure 13. TEM images of the **(a)** Pt_2Ag_1/CoP, **(b)** Pt_2Rh_1/CoP, **(c)** Pt_2Au_1/CoP and **(e)** Pt_2Pd_1/CoP catalysts at the metal loading of 1.0 *wt*%. The insets show the size distributions of the loaded metal nanoparticles.

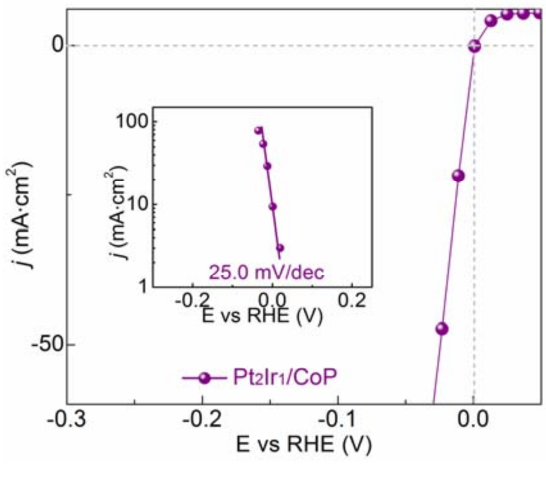

Supplementary Figure 14. HER performance of the Pt_2Ir_1/CoP catalysts in H_2-saturated 0.5 M H_2SO_4 electrolyte.

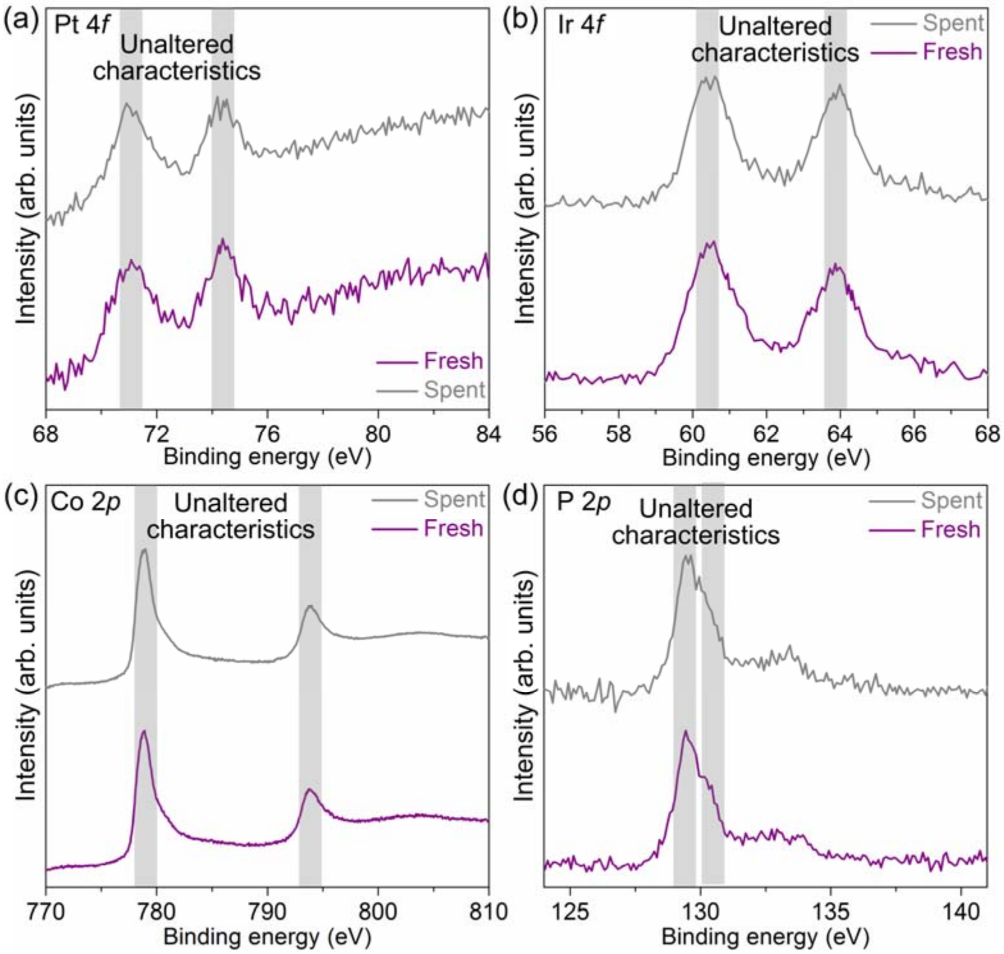

Supplementary Figure 15. High-resolution XPS spectra in **(a)** Pt 4*f*, **(b)** Ir 4*f*, **(c)** Co 2*p* and **(d)** P 2*p* region for the fresh and spent Pt_2Ir_1/CoP catalysts.

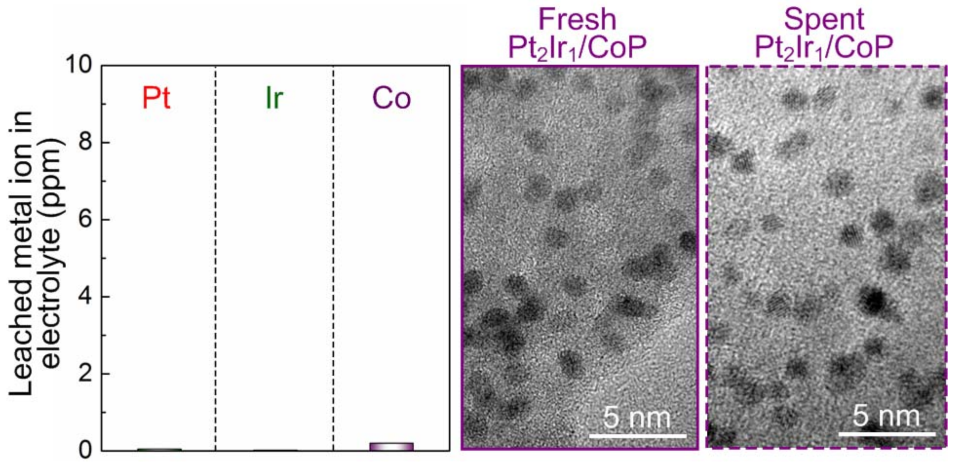

Supplementary Figure 16. Durability analysis for the Pt_2Ir_1/CoP catalysts. The ICP (left) and TEM (right) results show the negligible leaching of catalyst composition and morphology change during HER operation.

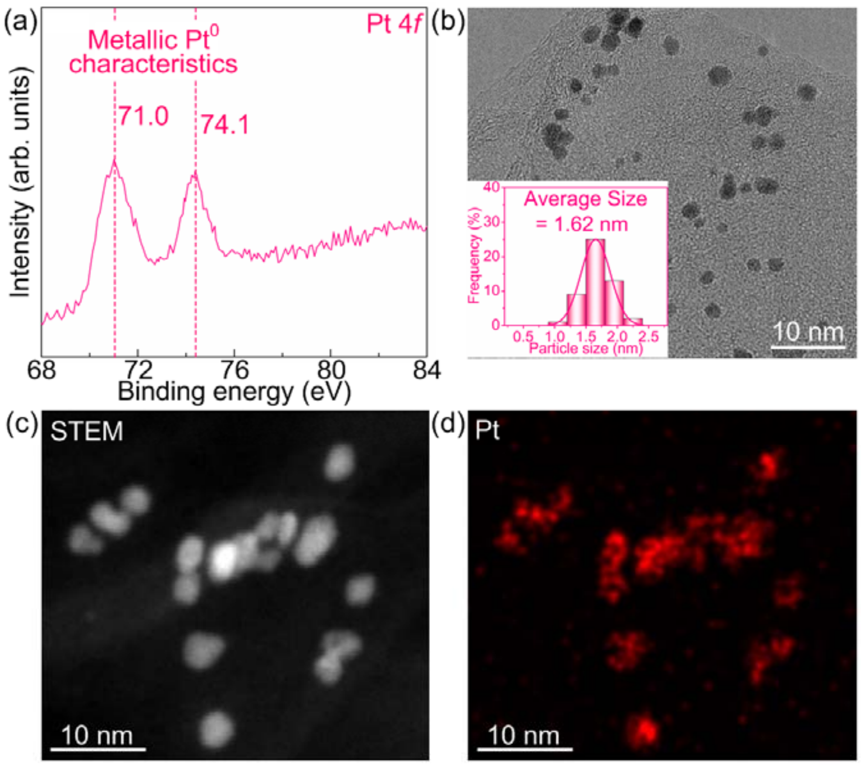

(e)

Catalyst	Catalyst mass (mg)	Constant volume (mL)	Measured Pt content (ppm)	Pt loading (*wt.* %)
Pt/rGO	50	100	5.3	1.05

Supplementary Figure 17. Characterizations of Pt/rGO. (a) High-resolution XPS spectrum in Pt 4*f* region. (b) TEM images. Inset is the size distribution of Pt. (c) STEM images and (d) EDX mapping. (e) ICP-MS analysis.

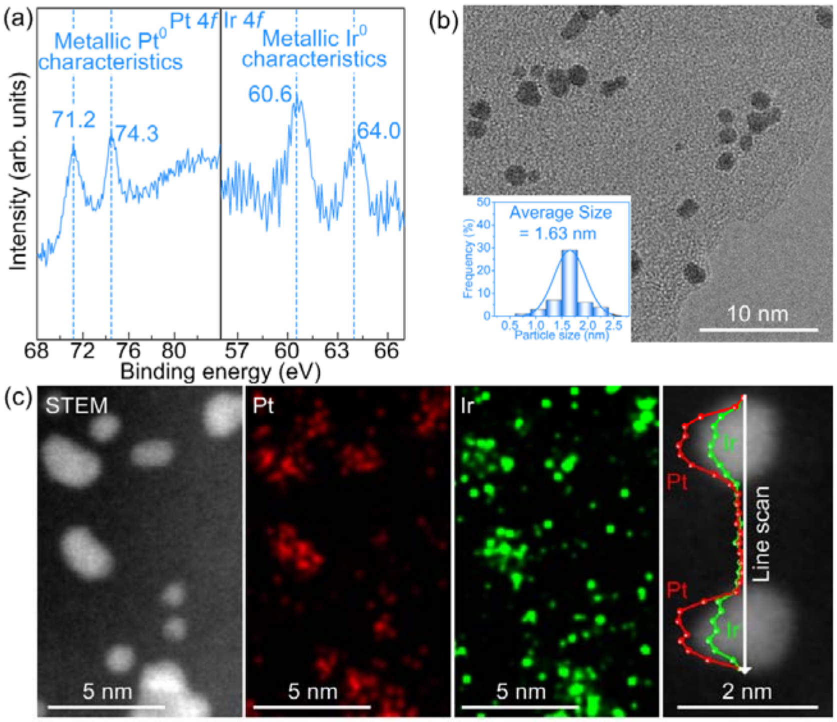

(d)

Catalyst	Catalyst mass (mg)	Constant volume (mL)	Measured Pt content (ppm)	Measured Ir content (ppm)	Molar ratio of Pt : Ir	Alloy loading (*wt.* %)
Pt_2Ir_1/rGO	50	100	3.6	1.8	1.99	1.06

Supplementary Figure 18. Characterizations of Pt_2Ir_1/rGO. (a) High-resolution XPS spectra in Pt 4*f* and Ir 4*f* region. (b) TEM image. Inset is the size distribution of Pt_2Ir_1. (c) STEM images, EDX mapping and line scan. (d) ICP-MS analysis.

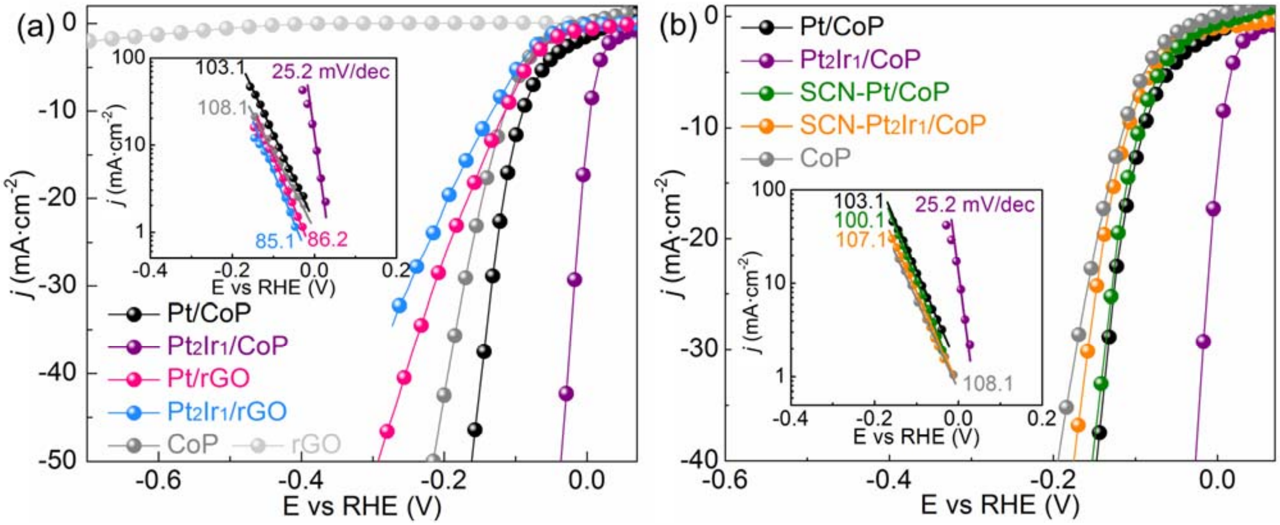

Supplementary Figure 19. Catalytic contribution analysis in Ar-saturated 0.5 M H_2SO_4. (a) HER performance of Pt/CoP, Pt_2Ir_1/CoP, Pt/rGO and Pt_2Ir_1/rGO. (b) HER performance of Pt/CoP and Pt_2Ir_1/CoP catalysts in the presence or absence of SCN^- probe.

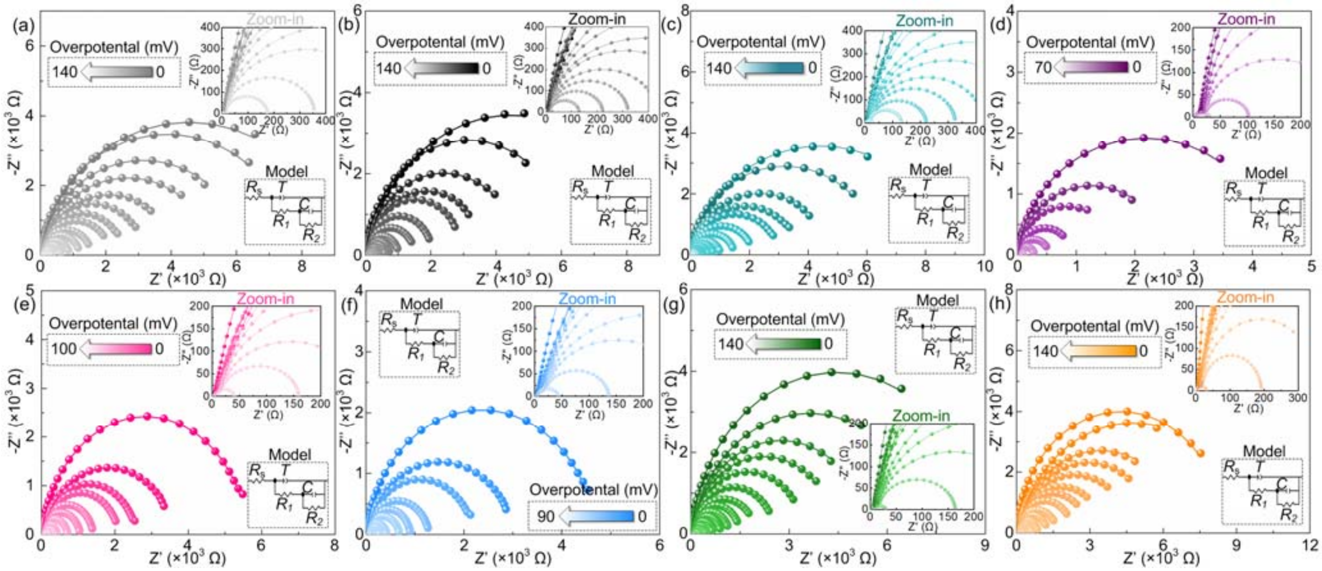

Supplementary Figure 20. Electrochemical impedance spectra analysis in Ar-saturated 0.5 M H_2SO_4. Nyquist plots for (a) bare CoP, (b) Pt/CoP, (c) Ir/CoP, (d) Pt_2Ir_1/CoP, (e) Pt/rGO, (f) Pt_2Ir_1/rGO, (g) SCN-Pt/CoP and (h) SCN-Pt_2Ir_1/CoP catalysts at various HER overpotentials. Zoom-in parts were correspondingly presented as inset. The scattered symbols represent the experimental results, and the solid lines are simulated fitting results. The inset also shows the equivalent circuit for the simulation. The fitted parameters are summarized in Table S7.

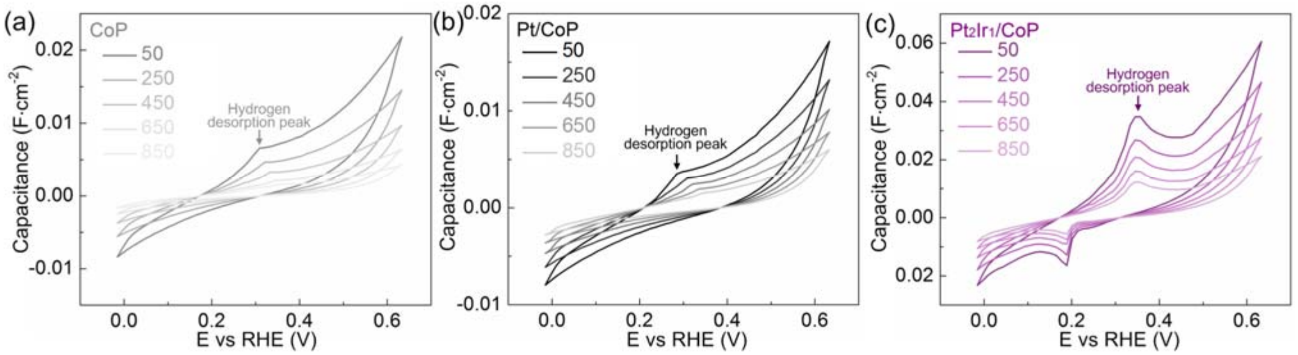

Supplementary Figure 21. Capacitance *vs.* voltage profiles obtained from cyclic voltammograms of (a) bare CoP, (b) Pt/CoP and (c) Pt_2Ir_1/CoP catalysts with the scan rate from 50 to 850 mV·s^{-1} in Ar-saturated 0.5 M H_2SO_4.

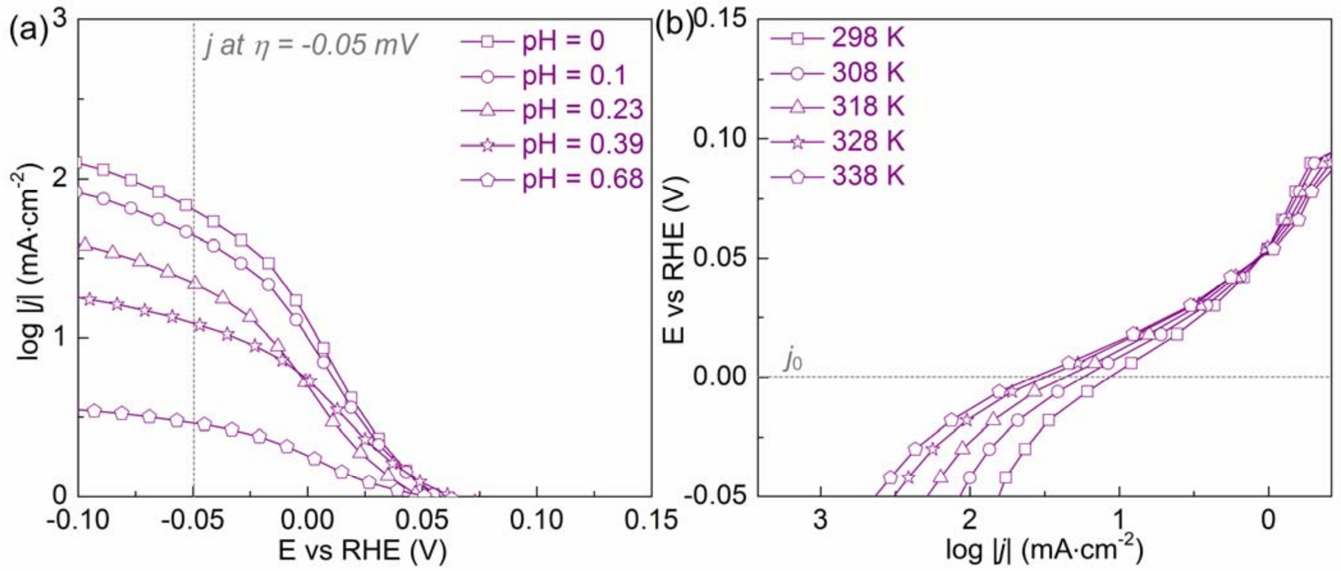

Supplementary Figure 22. (a) Tafel curves of Pt_2Ir_1/CoP catalysts in Ar-saturated H_2SO_4 with pH ranging from 0 to 0.68. **(b)** Tafel curves of Pt_2Ir_1/CoP catalysts in Ar-saturated 0.5 M H_2SO_4 at different temperatures ranging from 298 to 338 K.

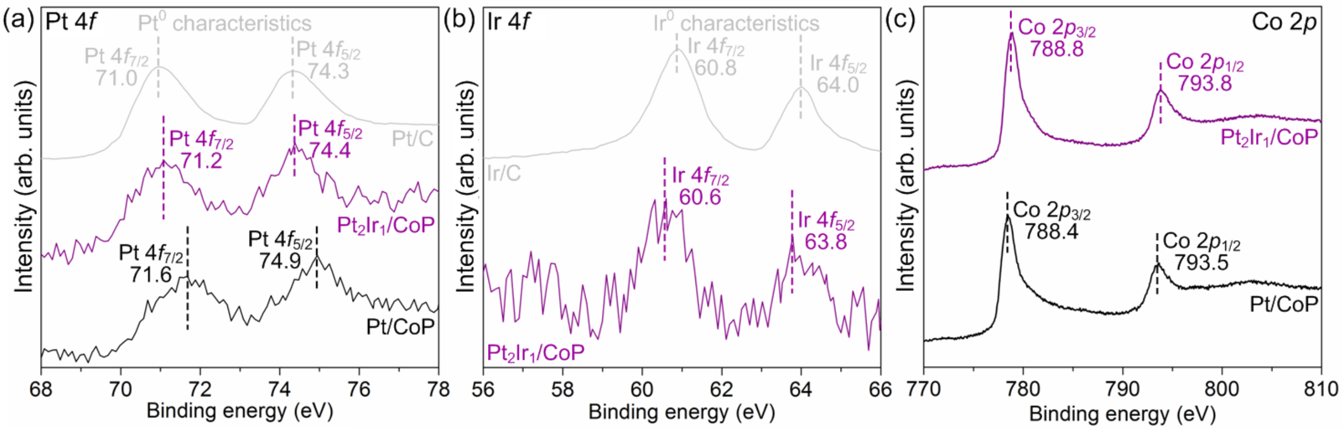

Supplementary Figure 23. High-resolution XPS spectra in (a) Pt 4*f*, (b) Ir 4*f* and (c) Co 2*p* region of the Pt/CoP and Pt_2Ir_1/CoP catalysts. Compared with Pt/CoP, the XPS peaks of Pt $4f_{7/2}$ and Pt $4f_{5/2}$ for Pt_2Ir_1/CoP shift to the low binding energy, while its XPS peaks of Co $2p_{3/2}$ and Co $2p_{2/1}$ shift to the high binding energy. In addition, the XPS peaks of Pt $4f_{7/2}$ (71.2 eV) and Pt $4f_{5/2}$ (74.4 eV) for Pt_2Ir_1/CoP is close to the characters of the Pt^0 (Pt $4f_{7/2}$ = 71.0 eV and Pt $4f_{5/2}$ = 74.3 eV) in Pt/C benchmark, while its XPS peaks of Ir $4f_{7/2}$ (60.6 eV) and Ir $4f_{5/2}$ (63.8 eV) is close to the characters of the Ir^0 (Ir $4f_{7/2}$ = 60.8 eV and Ir $4f_{5/2}$ = 64.0 eV) in Ir/C benchmark.

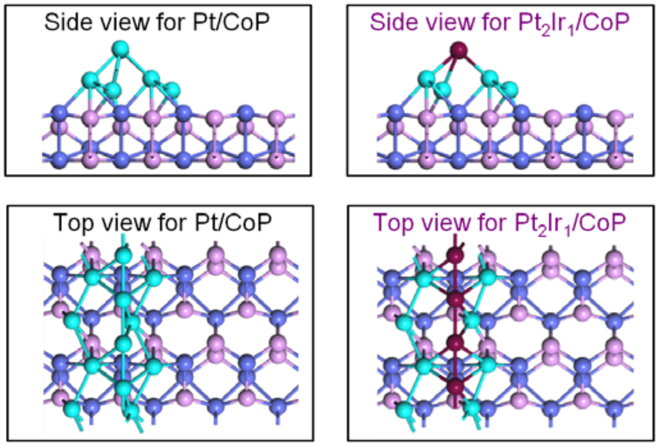

Supplementary Figure 24. Side and top view of Pt/CoP and Pt_2Ir_1/CoP surfaces.

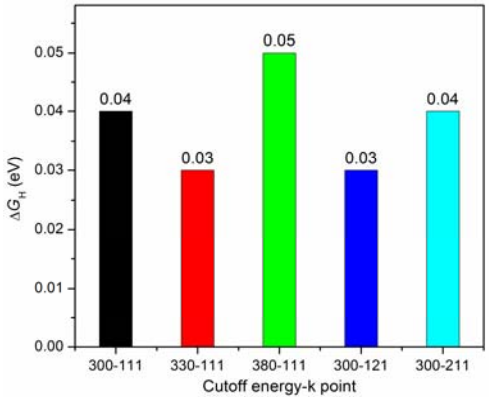

Supplementary Figure 25. The ΔG_{H} values of site 4 on Pt/CoP surface in different cutoff energy and k-point. The results show there are no obvious difference for the adsorption thermodynamics calculation in different cases.

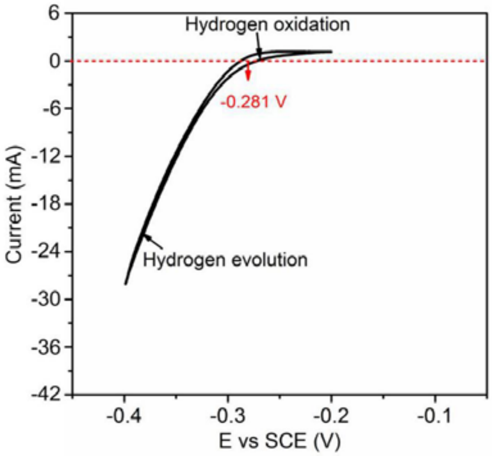

Supplementary Figure 26. The cyclic voltammetry result of RHE calibration in 0.5 M H_2SO_4, E(RHE) = E(SCE) + 0.281 V.

Supplementary Table 1. ICP-MS analysis of series of PtIr/CoP catalysts.

Catalyst	Alloy loading [wt.%]	Pt loading [wt.%]	Ir loading [wt.%]	Molar ratio of Pt : Ir
Pt_3Ir_1/CoP	1.03	0.77	0.26	2.97
Pt_2Ir_1/CoP	0.99	0.67	0.32	2.04
Pt_1Ir_1/CoP	1.01	0.51	0.50	0.99
Pt_1Ir_2/CoP	1.05	0.34	0.71	0.48
Pt_1Ir_3/CoP	1.03	0.25	0.78	0.31

Supplementary Table 2. ICP-MS analysis of the Pt_2Ir_1/CoP catalysts with different metal loading.

Catalyst	Alloy loading [wt.%]	Pt loading [wt.%]	Ir loading [wt.%]	Molar ratio of Pt : Ir
Pt_2Ir_1/CoP-0.5	0.47	0.32	0.15	2.09
Pt_2Ir_1/CoP-1.0	1.04	0.81	0.23	2.01
Pt_2Ir_1/CoP-1.5	1.51	1.0	0.51	1.97
Pt_2Ir_1/CoP-2.0	1.97	1.32	0.65	2.02

Supplementary Table 3. ICP-MS analysis of series of PtM/CoP catalysts.

Catalyst	Alloy loading [wt.%]	Pt loading [wt.%]	Foreign metal loading [wt.%]	Molar ratio of Pt :foreign metal
Pt_2Rh_1/CoP	1.05	0.83	0.22 (Rh)	1.98 (Pt : Rh)
Pt_2Pd_1/CoP	1.04	0.81	0.23 (Pd)	2.01 (Pt : Pd)
Pt_2Ag_1/CoP	1.0	0.78	0.22 (Ag)	2.0 (Pt : Ag)
Pt_2Au_1/CoP	0.98	0.64	0.34 (Au)	2.06 (Pt : Au)

Supplementary Table 4. Comparison of HER performance in acidic media for Pt_2Ir_1/CoP with the earth-abundant and noble metal-based HER catalysts, especially the single-atom Pt catalysts.

**Catalyst**	**Loading of noble metal [μg/cm^2]**	**Size of noble metal**	η_{20} **[mV]**	**Tafel slope [mV/dec]**	**Durability**	**Suppl. Ref.**
Pt_2Ir_1/CoP	0.6	~ 1.6 nm	7	25.2	500 h	This work
Ni_2P NPs	0	–	130	46	–	Ref. 7
CoP NWs	0	–	100	51	22 h	Ref. 8
Co-Fe-P 3D electrode	0	–	80	45	–	Ref. 9
Mo-W-P	0	–	101	52	8 h	Ref. 10
$Ni_{0.33}Co_{0.67}S_2$ NWs	0	–	88	44.1	< 3 h	Ref. 11
CoSP/CNTs	0	–	65	55	24 h	Ref. 12
CoPS NPs	0	–	65	56	35 h	Ref. 13
NiCoP/rGO	0	–	55	45.2	18 h	Ref. 14
PANI/CoP	0	–	70	34.5	30 h	Ref. 15
CoP/Co-MoF	0	–	33	43	17 h	Ref. 16
CoP_2	0	–	53	32	36 h	Ref. 17
$Pt_{tripods}$@PAA	79	~ 4 nm	0	26	1.67 h	Ref. 18
Pt/MoS_2	18	Atomic-size	>150	96	5000 cycles	Ref. 19
PtSA-NT-NF	140	Atomic-size	46	30	24 h	Ref. 20
ALD50Pt/NGNs	1.1	Atomic-size	54	29	1000 cycles	Ref. 21
Pt-GDY2	4.65	Atomic-size	92	46.6	2.78 h	Ref. 22
$Pt-MoS_2$	7.0	Atomic-size	47	25.0	35 h	Ref. 23
$AL-Pt/Pd_3Pb$	1.6	Atomic-size	17	18	25 h	Ref. 24
Pt $SA/m-WO_{3-x}$	0.86	Atomic-size	76	45	10 h	Ref. 25
Pt SASs/AG	31	Atomic-size	19	29.3	24 h	Ref. 26
$Mo_2TiC_2T_x–Pt_{SA}$	12	Atomic-size	43	30	100 h	Ref. 27
Pt_1/OLC	1.37	Atomic-size	55	36	100 h	Ref. 28
$Pt/Co_{0.85}Se$	204	Atomic-size	60	26	40 h	Ref. 29
$Rh-MoS_2$	24	Atomic-size	90	54	20 h	Ref. 30
$Pt-SA/MoO_x$	1.68	Atomic-size	18	123	20 h	Ref. 31
Pt-PVP/TNR	21.9	Atomic-size	27	27	44 h	Ref. 32
Pt/PCM	113	Atomic-size	142	65.3	5 h	Ref. 33
RuCoP	60	Atomic-size	25	31	150 h	Ref. 34
Ir@CON	500	~ 2.29 nm	16	27.0	10000 cy-cles	Ref. 35

Supplementary Table 5. Comparison of HER performance of Pt_2Ir_1/CoP with the previously reported HSBB electrocatalysts in acid media.

**Catalyst**	**Loading of noble metal [μg/cm^2]**	**η_{20}[mV]**	**Tafel slope [mV/dec]**	**Durability**	**Suppl. Ref.**
Pt_2Ir_1/CoP	0.6	7	25.2	500 h	This work
$Pt/RuCeO_2$	1.9	72	31	8 h	Ref. 36
Pt SA/m-WO_{3-x}	0.86	76	45	10 h	Ref. 25
Pt-WO_3	–	52	32.9	5000 cycles	Ref. 37
Rh/SiNW	56	81	24	138 h	Ref. 1
Ir/SiNW	60	22	20	14 h	Ref. 38
Rh/MoS_2	16	47	24	22.2	Ref. 2
Pt/WO_3	16	42	73	19.4 h	Ref. 39
WO_2/WS_2	0	100	54	3.1 h	Ref. 40
EG-Pt/CoP-1.5	1.5	21	42.5	100 h	Ref. 41

Supplementary Table 6. Comparison of HER activity in H_2-saturated 0.5 M H_2SO_4 electrolyte for Pt_2Ir_1/CoP with the state-of-the-art HER electrocatalysts, especially Pt-based catalysts.

**Catalyst**	**Loading of noble metal [μg/cm^2]**	**Size of noble metal**	η_{20} **[mV]**	**Tafel slope [mV/dec]**	**Suppl. Ref.**
Pt_2Ir_1/CoP	0.6	~ 1.6 nm	9	25.0	This work
Ni_2P NPs	0	–	130	46	Ref. 7
$Ni_{0.33}Co_{0.67}S_2$ NWs	0	–	88	44.1	Ref. 11
CoPS NPs	0	–	65	56	Ref. 13
B-doped CoP	0	–	58	50	Ref. 42
CoP/Co-MoF	0	–	33	43	Ref. 16
CoP_2	0	–	53	32	Ref. 17
AL-Pt/Pd_3Pb	1.6	Atomic-size	17	18	Ref. 24
Pt SA/m-WO_{3-x}	0.86	Atomic-size	76	45	Ref. 25
Pt_1/OLC	1.37	Atomic-size	55	36	Ref. 28
A-CoPt-NC	0.419	Atomic-size	32	31	Ref. 43
K_2PtCl_4@NC-M	5.6	Atomic-size	15	21	Ref. 44
Pt_1/MC	0.26	Atomic-size	32	26	Ref. 45
Pt-PVP/TNR	21.9	Atomic-size	27	27	Ref. 32
$Mo_2TiC_2T_x$–PtSA	12	Atomic-size	43	30	Ref. 27
Pt-MoS_2	7.0	Atomic-size	47	25.0	Ref. 46
Pt-WO_3	—	Atomic-size	52	32.9	Ref. 37
RuCoP	60	Atomic-size	25	31	Ref. 34
RuP_2@NPC	233	Atomic-size	59	38	Ref. 47

Supplementary Table 7. The fitted parameters of the EIS data of bare CoP, Pt/CoP, Ir/CoP, Pt_2Ir_1/CoP, Pt/rGO, Pt_2Ir_1/rGO, SCN-Pt/CoP and SCN-Pt_2Ir_1/CoP for HER.

Catalysts	η[mV]	R_s[Ω]	T[F s^{n-1}]	R_1[Ω]	n_1	R_2[Ω]	C_φ [F]
CoP	0	3.58	0.0041	21.1	0.86	9120	0.0018
	-10	3.68	0.0039	21.0	0.90	7762	0.0024
	-20	3.63	0.0043	20.9	0.87	6310	0.0026
	-30	3.61	0.0042	20.8	0.83	5370	0.0032
	-40	3.64	0.0045	20.4	0.81	4266	0.004
	-50	3.59	0.0044	19.8	0.90	3162	0.007
	-60	3.65	0.0042	17.7	0.91	2512	0.010
	-70	3.61	0.0044	16.7	0.85	2138	0.0132
	-80	3.66	0.0042	15.8	0.86	1549	0.0145
	-90	3.57	0.0041	15.0	0.88	1191	0.0172
	-100	3.61	0.0045	14.6	0.83	879	0.0203
	-110	3.63	0.0046	13.9	0.89	616	0.0224
	-120	3.60	0.0038	12.8	0.92	340	0.024
	-130	3.54	0.0045	12.0	0.91	160	0.026
	-140	3.61	0.0041	11.4	0.81	–	–
Pt/CoP	0	3.58	0.0058	24.1	0.82	8912	0.0015
	-10	3.62	0.0062	24.0	0.91	6310	0.0025
	-20	3.57	0.0061	23.7	0.87	4786	0.004
	-30	3.56	0.0058	24.0	0.86	3715	0.0063
	-40	3.55	0.006	23.1	0.9	3020	0.0093
	-50	3.51	0.0054	22.8	0.91	2042	0.013
	-60	3.56	0.0059	21.0	0.83	1514	0.016
	-70	3.50	0.0064	20.7	0.88	1000	0.017
	-80	3.51	0.0061	20.4	0.87	735	0.018
	-90	3.49	0.006	19.8	0.93	588	0.019
	-100	3.63	0.0064	19.2	0.91	410	0.02
	-110	3.62	0.0058	18.8	0.90	299	0.021
	-120	3.55	0.0051	18.3	0.87	201	0.022
	-130	3.59	0.0065	18.0	0.86	109	0.0222

	-140	3.60	0.0062	17.3	0.82	–	–
	0	3.52	0.0040	24.4	0.83	8886	0.0019
	-10	3.61	0.0042	24.1	0.91	6405	0.0025
	-20	3.62	0.0041	23.7	0.85	4766	0.0027
	-30	3.60	0.0043	23.4	0.86	3691	0.0031
	-40	3.62	0.0043	23.0	0.87	3002	0.0042
	-50	3.57	0.0046	22.9	0.85	2112	0.0071
	-60	3.58	0.0044	22.0	0.88	1466	0.0102
**Ir/CoP**	-70	3.63	0.0041	21.7	0.82	999	0.0145
	-80	3.51	0.0040	21.4	0.85	740	0.0190
	-90	3.54	0.0045	20.8	0.86	566	0.0218
	-100	3.62	0.0043	20.2	0.88	406	0.0235
	-110	3.60	0.0046	19.7	0.90	302	0.0245
	-120	3.58	0.0040	19.0	0.83	198	0.0255
	-130	3.57	0.0039	18.1	0.86	110	0.0266
	-140	3.56	0.0042	17.5	0.88	–	–
	0	3.42	0.0025	35.2	0.90	5623	0.0001
	-10	3.51	0.0031	34.7	0.81	3630	0.0002
	-20	3.50	0.0027	34.3	0.83	2570	0.0007
	-30	3.55	0.0039	34.2	0.83	2089	0.0016
	-40	3.53	0.0033	33.8	0.85	1380	0.0027
**Pt/C**	-50	3.51	0.0031	33.3	0.82	1023	0.0037
	-60	3.49	0.0028	32.9	0.85	676	0.0047
	-70	3.46	0.0037	32.1	0.92	389	0.0055
	-80	3.51	0.0036	31.6	0.91	239	0.0061
	-90	3.42	0.0035	31.0	0.90	125	0.0065
	-100	3.44	0.0038	30.4	0.92	–	–
	0	3.61	0.0055	25.1	0.91	8912	0.0018
	-10	3.58	0.0051	24.6	0.85	7244	0.0018
**SCN-Pt/CoP**	-20	3.62	0.0057	24.2	0.86	5495	0.0020
	-30	3.60	0.0049	24.1	0.86	4466	0.0028
	-40	3.65	0.0053	23.7	0.88	3467	0.0033

	-50	3.62	0.0051	23.2	0.87	2570	0.0054
	-60	3.58	0.0058	22.8	0.81	1995	0.0097
	-70	3.54	0.0057	22.2	0.89	1479	0.014
	-80	3.55	0.0056	21.7	0.88	1149	0.015
	-90	3.66	0.0054	21.2	0.92	891	0.018
	-100	3.67	0.0049	20.5	0.81	660	0.021
	-110	3.60	0.0052	20.0	0.80	457	0.023
	-120	3.59	0.0053	19.6	0.83	281	0.025
	-130	3.61	0.0058	19.2	0.86	141	0.026
	-140	3.67	0.0056	18.7	0.80	–	–
**Pt_2Ir_1/CoP**	0	3.52	0.0060	24.9	0.90	4365.2	0.007
	-10	3.51	0.0054	24.2	0.92	2512	0.019
	-20	3.55	0.0055	23.6	0.81	1621.8	0.051
	-30	3.56	0.0056	23.5	0.80	891.3	0.066
	-40	3.61	0.0057	23.0	0.85	512.9	0.081
	-50	3.62	0.0059	22.6	0.82	263.2	0.088
	-60	3.49	0.0062	22.0	0.81	77.6	0.096
	-70	3.50	0.0061	21.4	0.83	–	–
**Pt_2Ir_1/C**	0	3.51	0.0035	36.4	0.91	4677	0.0001
	-10	3.53	0.0030	35.9	0.82	3020	0.0007
	-20	3.54	0.0037	35.5	0.82	2188	0.0024
	-30	3.45	0.0029	35.4	0.82	1259	0.0037
	-40	3.46	0.0023	35.0	0.86	871	0.0048
	-50	3.55	0.0035	34.5	0.83	646	0.0058
	-60	3.57	0.0038	34.1	0.83	389	0.0065
	-70	3.53	0.0027	33.9	0.90	246	0.0069
	-80	3.50	0.0034	32.8	0.93	100	0.0072
	-90	3.47	0.0036	32.2	0.91	–	–
**SCN-Pt_2Ir_1/CoP**	0	3.42	0.0045	26.3	0.81	9549	0.0010
	-10	3.51	0.0041	25.8	0.95	8511	0.0012
	-20	3.52	0.0047	25.5	0.82	6918	0.0017
	-30	3.50	0.0039	25.3	0.81	5888	0.0020

-40	3.55	0.0043	24.9	0.80	4786	0.0038
-50	3.52	0.0055	24.4	0.82	3631	0.0064
-60	3.49	0.0049	23.9	0.83	2951	0.011
-70	3.44	0.0047	23.4	0.86	2290	0.013
-80	3.45	0.0046	22.9	0.82	1659	0.016
-90	3.56	0.0044	22.4	0.82	1258	0.019
-100	3.57	0.0047	21.7	0.91	851	0.021
-110	3.50	0.0051	21.2	0.90	616	0.024
-120	3.49	0.0050	20.8	0.93	338	0.025
-130	3.51	0.0052	20.2	0.85	169	0.026
-140	3.57	0.0051	19.6	0.90	–	–

Supplementary References:

1 Zhu, L. *et al.* A rhodium/silicon co-electrocatalyst design concept to surpass platinum hydrogen evolution activity at high overpotentials. *Nat. Commun.* **7**, 12272 (2016).

2 Cheng, Y. *et al.* Rh/MoS_2 Nanocomposite catalysts with Pt-like activity for hydrogen evolution reaction. *Adv. Funct. Mater.* **27**, 1700359 (2017).

3 Zhang, G., Zang, S. & Wang, X. Layered $Co(OH)_2$ deposited polymeric carbon nitrides for photocatalytic water oxidation. *ACS Catal.* **5**, 941-947 (2015).

4 Zhang, G. *et al.* Highly active and stable catalysts of phytic acid-derivative transition metal phosphides for full water splitting. *J. Am. Chem. Soc.* **138**, 14686-14693 (2016).

5 El Sawy, E. N., Handal, H. T., Thangadurai, V. & Birss, V. I. Pt_xIr_y alloy nanoparticles with fully tunable bulk and surface compositions. *J. Mater. Chem. A* **4**, 15400-15410 (2016).

6 Berlowitz, P. J., Peden, C. H. & Goodman, D. W. Kinetics of carbon monoxide oxidation on single-crystal palladium, platinum, and iridium. *J. Phys. Chem.* **92**, 5213-5221 (1988).

7 Popczun, E. J. *et al.* Nanostructured nickel phosphide as an electrocatalyst for the hydrogen evolution reaction. *J. Am. Chem. Soc* **135**, 9267-9270 (2013).

8 Tian, J., Liu, Q., Asiri, A. M. & Sun, X. Self-supported nanoporous cobalt phosphide nanowire arrays: an efficient 3D hydrogen-evolving cathode over the wide range of pH 0–14. *J. Am. Chem. Soc.* **136**, 7587-7590 (2014).

9 Tan, Y. *et al.* Versatile nanoporous bimetallic phosphides towards electrochemical water splitting. *Energy Environ. Sci.* **9**, 2257-2261 (2016).

10 Wang, X.-D. *et al.* Novel porous molybdenum tungsten phosphide hybrid nanosheets on carbon cloth for efficient hydrogen evolution. *Energy Environ. Sci.* **9**, 1468-1475 (2016).

11 Peng, Z., Jia, D., Al-Enizi, A. M., Elzatahry, A. A. & Zheng, G. From water oxidation to reduction: homologous Ni–Co based nanowires as complementary water splitting electrocatalysts. *Adv. Energy Mater.* **5**, 1402031 (2015).

12 Liu, W. *et al.* A highly active and stable hydrogen evolution catalyst based on pyrite-structured cobalt phosphosulfide. *Nat. Commun.* **7**, 10771 (2016).

13 Cabán-Acevedo, M. *et al.* Efficient hydrogen evolution catalysis using ternary pyrite-type cobalt phosphosulphide. *Nat. Mater.* **14**, 1245 (2015).

14 Li, J. *et al.* Mechanistic insights on ternary $Ni_{2-x}Co_xP$ for hydrogen evolution and their hybrids with graphene as highly efficient and robust catalysts for overall water splitting. *Adv. Funct. Mater.* **26**, 6785-6796 (2016).

15 Feng, J.-X., Tong, S.-Y., Tong, Y.-X. & Li, G.-R. Pt-like hydrogen evolution electrocatalysis on PANI/CoP hybrid nanowires by weakening the shackles of hydrogen ions on the surfaces of catalysts. *J. Am. Chem. Soc.* **140**, 5118-5126 (2018).

16 Liu, T. *et al.* CoP-doped MOF-based electrocatalyst for pH-universal hydrogen evolution reaction. *Angew. Chem. Int. Edit.* **58**, 4679-4684 (2019).

17 Li, H. *et al.* Phosphorus-rich colloidal cobalt diphosphide (CoP_2) Nanocrystals for electrochemical and photoelectrochemical hydrogen evolution. *Adv. Mater.* **31**, 1900813 (2019).

18 Xu, G.-R. *et al.* Polyallylamine-functionalized platinum tripods: enhancement of hydrogen evolution reaction by proton carriers. *ACS Catal.* **7**, 452-458 (2016).

19 Deng, J. *et al.* Triggering the electrocatalytic hydrogen evolution activity of the inert two-dimensional MoS_2 surface via single-atom metal doping. *Energy Environ. Sci.* **8**, 1594-1601 (2015).

20 Luo, J., Zhang, L., Han, L., Liu, H. & Liu, X. Potential-cycling synthesis of single Pt atoms for efficient hydrogen evolution in neutral media. *Angew. Chem. Int. Edit.* **56**, 13694 (2017).

21 Cheng, N. *et al.* Platinum single-atom and cluster catalysis of the hydrogen evolution reaction. *Nat. Commun.* **7** 13638 (2016).

22 Yin, X.-P. *et al.* Engineering the coordination environment of single-atom platinum anchored on graphdiyne for optimizing electrocatalytic hydrogen evolution. *Angew. Chem. Int. Edit.* **57**, 9382-9386 (2018).

23 Chen, Z. *et al.* Interface confined hydrogen evolution reaction in zero valent metal nanoparticles-intercalated molybdenum disulfide. *Nat. Commun.* **8**, 14548 (2017).

24 Yao, Y. *et al.* Engineering the electronic structure of submonolayer Pt on intermetallic Pd_3Pb via charge transfer boosts the hydrogen evolution reaction. *J. Am. Chem. Soc.* **141**, 19964-19968, (2019).

25 Park, J. *et al.* Investigation of the support effect in atomically dispersed Pt on WO_{3-x} for utilization of Pt in the hydrogen evolution reaction. *Angew. Chem. Int. Edit.* **58**, 16038-16042, (2019).

26 Ye, S. *et al.* Highly stable single Pt atomic sites anchored on aniline-stacked graphene for hydrogen evolution reaction. *Energy Environ. Sci.* **12**, 1000-1007 (2019).

27 Zhang, J. *et al.* Single platinum atoms immobilized on an MXene as an efficient catalyst for the hydrogen evolution reaction. *Nat. Catal.* **1**, 985-992 (2018).

28 Liu, D. *et al.* Atomically dispersed platinum supported on curved carbon supports for efficient electrocatalytic hydrogen evolution. *Nat. Energy* **4**, 512-518 (2019).

29 Jiang, K. *et al.* Single platinum atoms embedded in nanoporous cobalt selenide as electrocatalyst for accelerating hydrogen evolution reaction. *Nat. Commun.* **10**, 1743 (2019).

30 Meng, X. *et al.* Distance synergy of MoS_2-confined rhodium atoms for highly efficient hydrogen evolution. *Angew. Chem. Int. Edit.* **59**, 10502 (2020).

31 Xu, J. *et al.* Amorphous MoO_x-stabilized single platinum atoms with ultrahigh mass activity for acidic hydrogen evolution. *Nano Energy* **70**, 104529 (2020).

32 Li, C. *et al.* Polyvinylpyrrolidone-coordinated single-site platinum catalyst exhibits high activity for hydrogen evolution reaction. *Angew. Chem. Int. Edit.* **59**, 15902 (2020).

33 Zhang, H. *et al.* Dynamic traction of lattice-confined platinum atoms into mesoporous carbon matrix for hydrogen evolution reaction. *Sci. Adv.* **4**, eaao6657 (2018).

34 Xu, J. *et al.* Boosting the hydrogen evolution performance of ruthenium clusters through synergistic coupling with cobalt phosphide. *Energy Environ. Sci.* **11**, 1819-1827 (2018).

35 Mahmood, J. *et al.* Encapsulating iridium nanoparticles inside a 3D cage-like organic network as an efficient and durable catalyst for the hydrogen evolution reaction. *Adv. Mater.* **30**, 1805606, (2018).

36 Liu, T. *et al.* Selective loading of atomic platinum on a $RuCeO_x$ support enables stable hydrogen evolution at high current densities. *Angew. Chem. Int. Edit.* **59**, 20423 (2020).

37 Xie, C. *et al.* In-situ phase transition of WO_3 boosting electron and hydrogen transfer for enhancing hydrogen evolution on Pt. *Nano Energy* **71**, 104653 (2020).

38 Sheng, M. *et al.* Approaching the volcano top: iridium/silicon nanocomposites as efficient electrocatalysts for the hydrogen evolution reaction. *ACS Nano* **13**, 2786 (2019).

39 Tian, H. *et al.* Oxygen vacancy-assisted hydrogen evolution reaction of the Pt/WO 3 electrocatalyst. *J.Mater. Chem. A* **7**, 6285-6293 (2019).

40 Yang, L. *et al.* Synergistic $WO_3 \cdot 2H_2O$ nanoplates/WS_2 hybrid catalysts for high-efficiency hydrogen evolution. *ACS Appl. Mater. Inter.* **8**, 13966-13972 (2016).

41 Li, J. et al. Ethylene-glycol ligand environment facilitates highly efficient hydrogen evolution of Pt/CoP through proton concentration and hydrogen spillover. *Energy Environ. Sci.* **12**, 2298-2304 (2019).

42 Cao, E. *et al.* Boron-induced electronic-structure reformation of CoP nanoparticles drives enhanced pH-universal hydrogen evolution. *Angew. Chem. Int. Edit.* **59**, 4154-4160 (2020).

43 Zhang, L. *et al.* Charge polarization from atomic metals on adjacent graphitic layers for enhancing the hydrogen evolution reaction. *Angew. Chem. Int. Edit.* **58**, 9404-9408 (2019).

44 Jin, H. *et al.* Simple and scalable mechanochemical synthesis of noble metal catalysts with single atoms toward highly efficient hydrogen evolution. *Adv. Funct. Mater.* **30**, 2000531 (2020).

45 Wei, H. *et al.* Iced photochemical reduction to synthesize atomically dispersed metals by suppressing nanocrystal growth. *Nat. Commun.* **8**, 1-8 (2017).

46 Chen, Z. *et al.* Interface confined hydrogen evolution reaction in zero valent metal nanoparticles-intercalated molybdenum disulfide. *Nat. Commun.* **8**, 1-9 (2017).

47 Pu, Z., Amiinu, I. S., Kou, Z., Li, W. & Mu, S. RuP_2-based catalysts with platinum-like activity and higher durability for the hydrogen evolution reaction at all pH values. *Angew. Chem. Int. Edit.* **56**, 11559-11564 (2017).
